# The Benefits of Medical Group Construction for Healthcare Professionals: A Survey of Six Tightly Knit Pilot Urban Medical Groups

**DOI:** 10.3390/healthcare13222846

**Published:** 2025-11-10

**Authors:** Chong Tian, Yiyang Deng, Tian Gan, Xue Bai

**Affiliations:** School of Nursing, Tongji Medical College, Huazhong University of Science and Technology, Wuhan 430030, China; tianchong0826@hust.edu.cn (C.T.); m202375984@hust.edu.cn (Y.D.); m202375986@hust.edu.cn (T.G.)

**Keywords:** tightly knit urban medical group, healthcare professionals, personal development, career advancement, integrated healthcare reform

## Abstract

**Background/Objectives:** As part of China’s efforts to build a high-quality and efficient integrated healthcare delivery system, tightly knit urban medical groups (TKUMGs) have emerged as a key model for promoting inter-institutional collaboration. While existing studies have focused on organizational outcomes, limited empirical evidence is available regarding the personal benefits experienced by healthcare professionals within TKUMGs. **Methods**: This study evaluated 2200 healthcare professionals’ perceived benefits from TKUMG participation in six pilot medical groups across two Chinese cities to identify factors associated with variations in career development outcomes. **Results**: Three distinct latent classes were identified: (1) A Limited Growth Group (32.4%), with minimal improvement across all dimensions; (2) a Skill Recognition Group (35.6%), with improvements in recognition and expertise utilization but limited gains in compensation and promotion; and (3) a Comprehensive Growth Group (32.0%), with comprehensive improvements in all six areas. Higher levels of participation and more positive attitudes toward TKUMG construction were significantly associated with inclusion in the more advanced development groups. Other significant factors included age, educational attainment, institutional role (leading vs. member), and departmental affiliation. TKUMG construction has generated heterogeneous benefits for healthcare professionals. Active engagement and institutional environments play critical roles in shaping individual development trajectories. **Conclusions**: Despite limitations related to this study’s cross-sectional design and self-reported data, these findings offer valuable insights for policymakers aiming to design incentive mechanisms, optimize human resource allocation, and enhance the sustainability of integrated healthcare models in urban China.

## 1. Introduction

China has embarked on establishing a high-quality and efficient integrated healthcare system. Central to this effort is the medical consortium—a collaborative network integrating diverse healthcare institutions within defined regions. Since 2017, the government has vigorously promoted medical consortia, forming over 18,000 networks nationwide till December 2023 [1]. In urban areas, tertiary public hospitals lead these consortiums, uniting community health service centers, and specialized facilities under a unified governance model that emphasizes resource sharing and regional cooperation were promoted [2]. Building on this foundation, a pilot program for tightly knit urban medical groups (TKUMGs), sometimes referred to as closely knit urban medical groups in policy documents and the literature, was launched in 2023 [3]. These groups deepen integration by pooling resources and harmonizing management across primary, secondary, and tertiary care institutions, marking a new strategic phase in urban healthcare reform [3].

Healthcare professionals are the core providers and operational foundation of medical services. Their clinical expertise and frontline experience enhance service quality and foster system-wide innovation through collaborative practices and problem-solving. At the same time, their ongoing feedback supports the refinement of policies and governance mechanisms [4]. Despite the model’s potential, healthcare professionals face implementation challenges, such as compensation disparities, increased workloads, and the need to adapt to new workflows, all of which may diminish their motivation to engage fully [5]. However, the existing research on medical consortia—both in China and internationally—has predominantly focused on organizational outcomes such as resource allocation [6], integrated service delivery [7,8], patient outcomes [9], or specific clinical programs and their impact on professional capacities [10,11,12]. Little attention has been given to the individual-level benefits that healthcare professionals derive from participation. It is therefore essential to understand their perceptions of medical consortia and assess how participation shapes their career development.

This gap is especially salient in the context of TKUMGs, which institutionalize deeper integration through unified personnel management, consolidated financial control, and shared clinical pathways. This transformative structure is likely to reshape the entire professional ecosystem, creating new avenues for career advancement for some roles or departments, while potentially constraining opportunities for others, in a manner distinct from earlier models. Therefore, this study investigates healthcare professionals’ perceptions and benefit experiences within six pilot TKUMGs. We hypothesize that (1) significant heterogeneity exists in benefit perceptions across different subgroups of healthcare professionals, particularly between those in leading versus member institutions, and among different departmental affiliations and (2) active participation in and positive attitudes toward TKUMGs are associated with greater perceived career benefits. The findings aim to provide empirical evidence for optimizing incentive mechanisms and promoting sustainable engagement in integrated healthcare reforms.

## 2. Materials and Methods

### 2.1. Study Design and Data Collection

This study employed a structured questionnaire to collect data from healthcare professionals in six pilot TKUMGs located in Qiqihar and Hefei, two cities that were among the first cities to implement the TKUMG pilot program in 2023 and that represent diverse geographic and economic contexts in northern and eastern China, respectively. Information on each TKUMG’s organizational structure and development was obtained from its construction plans and official evaluation reports. In accordance with the National Health Commission of China’s criteria for assessing urban medical groups, we evaluated each consortium across four domains—coordination of responsibilities and authorities, resource integration, operational collaboration, and governance mechanisms. Each domain was rated on a 10-point scale, yielding a maximum composite score of 40 for the overall assessment of consortium development (details were provided in Table A1 in the Appendix A.1). The TKUMG performance scores were assessed by two independent evaluators.

The questionnaire comprised three sections. (1) Demographics and Professional Background: Collected personal and workplace information, including gender, age, educational attainment, professional title, affiliated institution and its role within the TKUMG (lead vs. member), and department type. (2) Participation and Attitudes Toward the TKUMG: Seven items measured the extent of each professional’s involvement in TKUMG activities—such as patient referrals, inter-institutional consultations, training sessions, and collaborative research—and three items assessed their overall perceptions, recognition of the TKUMG’s impact on institutional development, and willingness to share expertise. Responses in both subscales were scored and then standardized so that each dimension had a maximum of 10 points. (3) Perceived Changes in Personal Development: Assessed self-reported changes across six career dimensions following TKUMG establishment: compensation level, workload, professional title advancement opportunities, training availability, patient recognition and trust, and the utilization of one’s skills and expertise. Each dimension was rated on a three-point scale (“decrease” = 1, “no change” = 2, and “increase” = 3). The three-point scale was selected for its simplicity and clarity in capturing directional changes, reducing cognitive burden on respondents [13,14]. The questionnaire was developed based on a literature review and expert consultation. A pilot test was conducted with 192 healthcare professionals from five cities (Wuhan, Yichang, Xiaogan, Zhijiang, and Shenzhen), and exploratory factor analysis supported the construct validity of the six career development dimensions, with a Cronbach’s alpha of 0.815 in the point study. The specific variable assignment after organizing the data collected from the three sections of the questionnaire were shown in Table 1.

### 2.2. Sampling and Recruitment

A cross-sectional survey was conducted among healthcare professionals from all six pilot TKUMGs in Qiqihar and Hefei. The sampling frame included all clinically active physicians, nurses, and allied health personnel employed within the leading or member institutions of these groups during the study period (April 2024–May 2024). Inclusion criteria were as follows: (1) full-time employment at a TKUMG-affiliated institution and (2) direct involvement in patient care or clinical support services. Temporary staff, administrative personnel without clinical duties, and those on long-term leave were excluded.

Recruitment was initiated through official institutional announcements and internal communication channels within each TKUMG. Participation was voluntary, and no incentives were provided. An electronic questionnaire was distributed via the “Wenjuanxing” platform (https://www.wjx.cn/, accessed on 1 April 2024). While the exact number of individuals who received the invitation could not be tracked due to the dissemination method and privacy protections, the total estimated eligible population across the six TKUMGs was approximately 16,063 healthcare professionals, based on staffing reports and institutional profiles. A total of 2200 complete responses were received. All participants were informed of the study’s purpose and provided electronic informed consent. Anonymity was assured, and IP addresses were used solely for preventing duplicate submissions and were not linked to individual identities.

### 2.3. Quality Control

To ensure data integrity, the “Wenjuanxing” platform was configured to enforce several control measures: (1) all questions were set as mandatory, requiring completion before submission and (2) only one submission per IP address or device was permitted to avoid duplicates. These measures, while crucial for data quality, also rendered it impossible to calculate a precise response rate, as the number of staff who saw the invitation but did not participate could not be determined. After the survey closed, the raw dataset was exported and screened by two independent researchers. They removed any responses that exhibited obvious patterns of invalidity (e.g., identical answers across all items, unrealistically short completion time). Any discrepancies in their assessments were resolved through discussion.

### 2.4. Statistical Analysis

Data entry and cleaning were performed using Excel 23.0. Descriptive analyses and group comparisons were conducted using SPSS 27.0, and latent class analysis (LCA) was carried out in Mplus 8.3. For descriptive statistics, continuous variables with a normal distribution are presented as mean ± standard deviation (SD), whereas non-normally distributed variables are reported as median and interquartile range (IQR). Categorical variables are summarized by frequencies and percentages. Responses to the six personal development items (compensation level, workload, career-advancement opportunities, training opportunities, patient recognition and trust, and utilization of expertise) were coded as “decrease” = 1, “no change” = 2, and “increase” = 3. LCA models with one to multiple latent classes were sequentially estimated. Model fit was assessed via the Akaike Information Criterion (AIC), Bayesian Information Criterion (BIC), sample-size-adjusted BIC (aBIC), the entropy index, the bootstrap likelihood ratio test (BLRT), and the Vuong– Lo–Mendell–Rubin test (VLMR). The optimal class solution was chosen based on these indices and on class assignment probabilities, excluding any class with a prevalence below 10%. For univariate analyses of post-TKUMG development, we applied chi-square tests for categorical variables and Kruskal–Wallis H tests for ordinal variables. A multinomial logistic regression model was then used to identify factors associated with latent class membership, with statistical significance set at α = 0.05.

## 3. Results

### 3.1. Medical Consortium Construction and Grouping

The six TKUMGs received comprehensive construction evaluation scores (out of 40) as follows: 3.87, 8.11, 15.56, 19.96, 22.02, and 28.25. Based on these values, the groups were categorized into three performance tiers: the high-score group (score > 20, *n* = 2), the medium-score group (score 10–20, *n* = 2), and the low-score group (score ≤ 10, *n* = 2) (Table 2).

### 3.2. Participant Demographics

A total of 2200 healthcare professionals from six pilot TKUMGs completed the survey. As shown in Table A2 in the Appendix A.2, their distribution across the groups was as follows: 19.5%, 34.1%, 23.0%, 14.8%, 7.5%, and 1.2%, respectively. Of these respondents, 942 individuals (42.8%) worked in the leading institutions, and 1258 (57.2%) worked in member institutions; 1558 participants (70.8%) were female. The age distribution was as follows: 321 participants (14.6%) were ≤30 years, 992 (45.1%) were between 31 and 40 years, 674 (30.6%) were between 41 and 50 years, and 213 (9.7%) were >50 years. Their educational background showed that 398 participants (18.1%) had an associate degree or lower, 1432 (65.1%) had a bachelor’s degree, and 370 (16.8%) had a graduate degree or higher. In terms of department affiliation, five departments, neurology, cardiology, oncology, endocrinology, and geriatrics, were specifically selected for their highly involvement in TKUMG activities. At last, 781 participants (35.5%) were from the five selected clinical departments; 533 (24.2%) were from general departments; 543 (24.7%) were from auxiliary departments like pathology, radiology, and ECG; and 343 (15.6%) were from other departments. Regarding professional titles, 129 participants (5.9%) had no title, 625 (28.4%) were at the junior level, 945 (43.0%) were at the intermediate level, 369 (16.8%) were at the senior level, and 132 (6.0%) were at the highest level. Differences were tested among the studies’ participants from different institution types within the TKUMGs regarding gender, age, education, professional title, affiliated TKUMG and department, individual attitude and individual participation (Table 3).

### 3.3. Personal Participation and Attitude

The overall score for individual participation in the activities related to the compact urban medical group was 5.00 (2.50, 8.75), while the score for personal attitudes towards the construction of the medical group was 10.00 (10.00, 10.00) (both with a maximum score of 10.00).

### 3.4. Latent Classes for Changes in Personal Development After the Construction of TKUMGs

We applied a latent class analysis (LCA) to six post-TKUMG development indicators—compensation, workload, promotion opportunities, training availability, patient recognition and trust, and utilization of expertise. Models with one through five classes were estimated sequentially. We stopped the model fitting when it reached the model of five latent classes because the model’s fit indices and the Bayesian Information Criterion (BIC) value stopped decreasing, indicating that the model fitting had reached the optimal state. Although, the model with four classes had the smallest BIC value and significant Vuong–Lo–Mendell–Rubin (VLMR, *p* < 0.001) and bootstrap likelihood ratio tests (BLRT, *p* < 0.001) in the model testing (i.e., a model with k classes is better than a model with k–1 class), considering that the model with four classes included a negligible class (3.5% of the sample) (Table 4). Therefore, the model with three classes was selected as it performed best comprehensively in terms of the model fit indices (AIC, BIC, and aBIC), model testing (BLRT, VLMR) and model characteristics (number of classes, the size of the smallest class, and class separation-measured entropy) [15]. In addition, Mplus uses multiple random starts to demonstrate the sufficient replication of the maximum likelihood for determining the best-fitting model, the software output indicated that the three-class model’s log-likelihood value reached a stable and optimal solution before hitting the maximum number of iterations of the software (default for Mplus is 100 times). This confirms that this model converged successfully and is robust [16].

Class 1: “Limited Growth Group” (32.4%): low probabilities of improvement across all six dimensions, indicating stagnant career development.

Class 2: “Skill Recognition Group” (35.6%): notable gains in patient recognition and expertise utilization but limited increases in compensation and promotion, reflecting an imbalance in benefits.

Class 3: “Comprehensive Growth Group” (32.0%): high probabilities of improvement across every dimension, signifying comprehensive career development.

**Figure 1:**
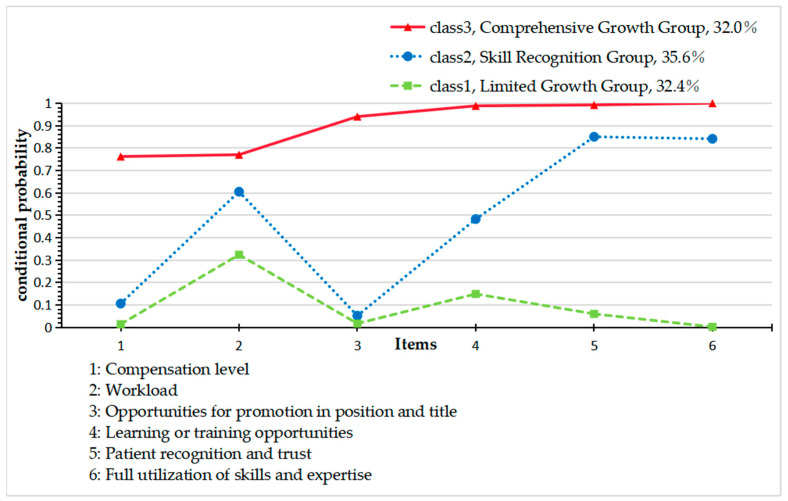
Conditional probability distribution diagram of three types of participants. The bar chart illustrates the three distinct subgroups of physicians identified by latent class analysis. Each class is characterized by its unique response pattern across six key work-related dimensions. The classes are labeled as follows: Class1 “Limited Growth Group” (32.4%)—those with lower overall growth and recognition, Class2 “Skill Recognition Group” (35.6%)—those that acknowledged improvements in patient trust and skill use, and Class3 “Comprehensive Growth Group” (32.0%)—those that perceived development across all areas.

The proportions and characteristics of these classes are illustrated in Figure 1.

### 3.5. Logistic Regression Analysis of Factors Influencing Healthcare Professionals’ Personal Development Changes After the Establishment of TKUMGs

The study participants were divided into two groups based on their institution type within the TKUMGs: the leading hospital group (*n* = 942) and the member institution group (*n* = 1258). The three latent categories identified from the latent class analysis were used as the dependent variable, while variables showing statistically significant differences in the univariate analysis were selected as independent variables for the multinomial logistic regression analysis (Table A2). After performing the parallel lines test (*p* < 0.05), unordered multinomial logistic regression models were used for group-wise analysis.

#### 3.5.1. Influencing Factors of Healthcare Professionals’ Personal Development Changes After the Establishment of TKUMGs in Leading Hospitals (Table 5)

- C2 Skill Recognition Group vs. C1 Limited Growth Group

Healthcare professionals aged 41–50 years were less likely to belong to the C2 Skill Recognition Group than those aged >50 years, with an OR of 0.439 (95% CI = 0.197–0.957). In contrast, higher participation levels increased the likelihood of being in the C2 Skill Recognition Group compared to the C1 Limited Growth Group (OR = 1.257, 95% CI = 1.153–1.245). A more positive attitude was also significantly associated with membership in the C2 Skill Recognition Group rather than the C1 Limited Growth Group (OR= 1.211, 95% CI = 1.099–1.335). Professionals in high-score TKUMGs had a higher likelihood of being in the C2 Skill Recognition Group compared to those in low-score groups (OR = 1.508, 95% CI = 1.019–2.232). In comparison to professionals in ancillary departments, professionals in other departments were less likely to belong to the C2 Skill Recognition Group (OR = 0.241, 95% CI = 0.100–0.580).

- C3 Comprehensive Growth Group vs. C1 Limited Growth Group

Higher levels of individual participation (OR= 1.406, 95% CI = 1.316–1.503) and more positive individual attitudes (OR= 4.926, 95% CI = 2.954–8.213) significantly increased the likelihood of being in the C3 Comprehensive Growth Group compared to the C1-Limited Growth Group. Compared to professionals in ancillary departments, professionals in general departments had a lower likelihood of belonging to the C3 Comprehensive Growth Group (OR = 0.398, 95% CI = 0.161–0.982), and professionals in other departments were even less likely to be in the C3 Comprehensive Growth Group (OR = 0.134, 95% CI = 0.040–0.447).

- C3 Comprehensive Growth Group vs. C2 Skill Recognition Group

Healthcare professionals aged 41–50 years were more likely to belong to the C3 Comprehensive Growth Group compared to those aged >50 years, with an OR of 2.241 (95% CI = 1.060–4.741). Higher participation levels were positively associated with being in the C3 Comprehensive Growth Group compared to the C2 Skill Recognition Group (OR =1.119, 95% CI = 1.058–1.184). A more positive attitude was significantly related to being in the C3 Comprehensive Growth Group rather than the C2 Skill Recognition Group, with an OR of 4.067 (95% CI = 2.446–6.763). Healthcare professionals in high-score TKUMGs were more likely to be in the C3 Comprehensive Growth Group compared to those in low-score TKUMGs, with an OR of 2.244 (95% CI = 1.442–3.493). However, those in medium-score groups had a lower likelihood of being in the C3 Comprehensive Growth Group compared to the C2 Skill Recognition Group, with an OR of 0.502 (95% CI = 0.345–0.731). Compared to ancillary departments, professionals in general departments had a lower likelihood of being in the C3 Comprehensive Growth Group (OR= 0.425, 95% CI = 0.192–0.942).

**Table 5:** Determinants of latent class membership for healthcare professionals’ development in leading hospital staff (*n* = 942).

Comparison Group	Variable/Category	β ^2^	SE ^2^	Wald χ^2^	*p*-Value	OR ^2^ (95% CI ^2^)
**C3 ^1^ (Ref: C1 ^1^)**	**Individual participation level**	0.341	0.034	101.841	<0.001	1.406 (1.316–1.503)
	**Individual attitude**	1.594	0.261	37.368	<0.001	4.926 (2.954–8.213)
	**Institution department** (ref: ancillary)					
	General departments	−0.922	0.461	3.993	0.046	0.398 (0.161–0.982)
	Other	−2.007	0.613	10.722	0.001	0.134 (0.040–0.447)
**C2 ^1^ (Ref: C1 ^1^)**	**Age** (ref: >50 years)					
	41–50 years	−0.824	0.407	4.087	0.043	0.439 (0.197–0.957)
	**Individual participation level**	0.228	0.031	55.934	<0.001	1.257 (1.153–1.245)
	**Individual attitude**	0.192	0.050	14.873	<0.001	1.211 (1.099–1.335)
	**TKUMG’s score** ^3^ (ref: low)					
	High-score group	0.411	0.200	4.222	0.040	1.508 (1.019–2.232)
	**Institution department** (ref: ancillary)					
	Others	−1.422	0.447	10.112	0.001	0.241 (0.100–0.580)
**C3 ^1^ (Ref: C2 ^1^)**	**Age** (Ref: >50 years)					
	41–50 years	0.807	0.382	4.459	0.035	2.241 (1.060–4.741)
	**Individual participation level**	0.113	0.029	15.238	<0.001	1.119 (1.058–1.184)
	**Individual attitude**	1.403	0.259	29.235	<0.001	4.067 (2.446–6.763)
	**TKUMG’s score** ^3^ (ref: low)					
	High-score group	1.098	0.336	10.666	0.001	2.244 (1.442–3.493)
	Medium-score group	−0.689	0.192	12.896	<0.001	0.502 (0.345–0.731)
	**Institution department** (ref: ancillary)					
	General departments	−0.855	0.406	4.444	0.035	0.425 (0.192–0.942)

Logistic regression models were developed to test the associations between demographic/professional factors and latent class membership. ^1^ C1 = C1-Limited Growth Group, C2 = C2-Skill Recognition Group, and C3 = C3-Comprehensive Growth Group. ^2^ Abbreviations: β = unstandardized coefficient; SE = standard error; OR = odds ratio; and CI = confidence level. ^3^ TKUMG’s score: the score of the overall assessment of TKUMG development above, and it is divided into high-score group, medium-score group, and low-score group according to the score.

#### 3.5.2. Influencing Factors of Healthcare Professionals’ Personal Development Changes After the Establishment of TKUMGs in Member Institutions (Table 6)

- C2 Skill Recognition Group vs. C1-Limited Growth Group

Higher levels of individual participation (OR = 1.182, 95% CI = 1.125–1.242) and more positive attitudes (OR = 1.216, 95% CI = 1.135–1.302) were significantly associated with an increased likelihood of belonging to the C2 Skill Recognition Group compared to the C1- Limited Growth Group.

- C3 Comprehensive Growth Group vs. C1-Limited Growth Group

Compared to individuals with a postgraduate degree, those with an undergraduate degree (OR = 2.260, 95% CI = 1.092–4.674) or a college education or lower (OR= 3.693, 95% CI = 1.653–8.254) were significantly more likely to belong to the C3 Comprehensive Growth Group. Additionally, higher individual participation (OR =1.407, 95% CI = 1.324–1.494) and more positive attitudes (OR = 2.701, 95% CI = 1.957–3.729) were strongly associated with an increased likelihood of belonging to the C3 Comprehensive Growth Group compared to the C1-Limited Growth Group. Healthcare professionals in the five selected clinical departments had a higher likelihood of being in the C3 Comprehensive Growth Group (OR = 2.918, 95% CI = 1.277–6.667) compared to those in ancillary departments. In contrast, professionals in general and other departments had a lower likelihood of belonging to the C3 Comprehensive Growth Group, with ORs of 0.399 (95% CI = 0.206–0.770) and 0.384 (95% CI = 0.201–0.736), respectively.

- C3 Comprehensive Growth Group vs. C2 Skill Recognition Group

Individuals aged ≤30 years were more likely to belong to the C3 Comprehensive Growth Group compared to those aged >50 years (OR = 1.923, 95% CI = 1.015–3.643). Higher individual participation (OR = 1.190, 95% CI = 1.129–1.254) and a more positive attitude (OR = 2.222, 95% CI = 1.611–3.065) increased the likelihood of being in the C3 Comprehensive Growth Group compared to the C2 Skill Recognition Group. Healthcare professionals in the five selected clinical departments had a higher likelihood of belonging to the C3 Comprehensive Growth Group compared to those in ancillary departments (OR = 2.221, 95% CI = 1.121–4.400). However, those in general departments and other departments were less likely to belong to the C3 Comprehensive Growth Group, with ORs of 0.448 (95% CI = 0.248–0.812) and 0.472 (95% CI = 0.261–0.855), respectively.

**Table 6:** Determinants of latent class membership for healthcare professionals’ development in member institution staff (*n* = 1258).

Comparison Group	Variable/Category	β ^2^	SE ^2^	Wald χ^2^	*p*-Value	OR ^2^ (95% CI ^2^)
**C3 ^1^ (Ref: C1 ^1^)**	**Education** (ref: postgraduate)					
	College or below	1.307	0.410	10.141	0.001	3.693 (1.653–8.254)
	Undergraduate	0.815	0.371	4.831	0.028	2.260 (1.092–4.674)
	**Individual participation level**	0.341	0.031	123.505	<0.001	1.407 (1.324–1.494)
	**Individual attitude**	0.994	0.164	36.483	<0.001	2.701 (1.957–3.729)
	**Institution department** (ref: ancillary)					
	The five selected clinical departments	1.071	0.422	6.449	0.011	2.918 (1.277–6.667)
	General departments	−0.920	0.336	7.495	0.006	0.399 (0.206–0.770)
	Other	−0.957	0.332	8.324	0.004	0.384 (0.201–0.736)
**C2 ^1^ (Ref: C1 ^1^)**	**Individual participation level**	0.167	0.025	43.699	<0.001	1.182 (1.125–1.242)
	**Individual attitude**	0.195	0.035	31.302	<0.001	1.216 (1.135–1.302)
**C3 ^1^ (Ref: C2 ^1^)**	**Age** (ref: >50 years)					
	≤30 years	0.654	0.326	4.017	0.045	1.923 (1.015–3.643)
	**Individual participation level**	0.174	0.027	42.061	<0.001	1.190 (1.129–1.254)
	**Individual attitude**	0.798	0.164	23.678	<0.001	2.222 (1.611–3.065)
	**Institution department** (ref: ancillary)					
	The five selected clinical departments	0.798	0.349	5.233	0.022	2.221 (1.121–4.400)
	General departments	−0.802	0.303	7.001	0.008	0.448 (0.248–0.812)
	Other	−0.750	0.303	6.141	0.013	0.472 (0.261–0.855)

Logistic regression models were performed to test the associations between demographic/professional factors and latent class membership. ^1^ C1 = C1-Limited Growth Group, C2 = C2-Skill Recognition Group, and C3 = C3-Comprehensive Growth Group. ^2^ Abbreviations: β = unstandardized coefficient; SE = standard error; OR = odds ratio; and CI = confidence level.

## 4. Discussion

The results of this study revealed the varying experiences of healthcare professionals within TKUMGs. While some professionals benefit significantly across multiple aspects of their career, others face barriers in compensation and advancement opportunities despite improvements in recognition and expertise utilization. Factors such as individual participation, attitude, department type, age, and medical group performance all played significant roles in shaping the personal development of healthcare professionals.

The LCA revealed three distinct classes of professional development among healthcare professionals following TKUMG implementation. These classes can be interpreted through the lens of the Career Construction Theory, which emphasizes how individuals adapt to changing organizational environments through different patterns of career behavior. The Class 1 Limited Growth Group, showing minimal improvement across all dimensions, may represent professionals who either lack the adaptive resources to navigate the new system or perceive insufficient organizational support to engage meaningfully with TKUMG opportunities. Identifying the causes of this stagnation (e.g., lack of support, opportunities, or resources) is crucial for addressing these challenges and enhancing the overall impact of medical group construction [17,18]. In contrast, the Class 2 Skill Recognition Group, which experienced improvements in patient recognition and expertise utilization but limited gains in compensation or promotion, exemplifies a partial adaptation pattern where professional competence is acknowledged without corresponding structural rewards. This disparity aligns with Social Exchange Theory principles, suggesting an imbalance between professional contributions and organizational reciprocation in terms of tangible career advancements. The Class 3 Comprehensive Growth Group, demonstrating across-the-board improvements, likely consists of individuals who have successfully leveraged TKUMG resources while receiving adequate organizational support, creating a virtuous cycle of professional development as predicted by both Social Exchange and Self-Determination theories.

The significant association between TKUMGs’ evaluation score and career development opportunities underscores the importance of organizational-level factors in shaping individual outcomes. This finding resonates with the Conservation of Resources Theory, which posits that individuals in better-resourced environments are better positioned to acquire new resources and achieve growth. When comparing leading and member institutions, professionals in member institutions were more likely to belong to the C1-Limited Growth Group and C2 Skill Recognition Group. A primary goal of TKUMGs is to enhance the service capabilities at the grassroots level [19]. The increased opportunities for learning and training felt by the medical staff of the member institutions, as well as the increased recognition from patients, reflect the achievements of the TKUMGs in this regard. However, the overall sense of benefit felt by employees in member institutions was not as high as that for the employees working in leading hospitals. This disparity may stem from two interrelated factors. First, as the formulator of the rules and the main promoter of the group’s construction, the leading hospital may take the interests of their own employees more into account in various systems of the group, thus occupying a certain advantageous position. In contrast, member institutions generally have less influence over the rules and operations within the group [20]. Additionally, primary care institutions—acting as member institutions—are typically level-one public welfare organizations, which operate under a dual-line management system for income and expenditure. While expenditures are funded by the national treasury, income is submitted to the state treasury [21]. This financial framework ensures the stability of funding sources for the institution but restricts their capacity to incentivize staff through financial means [22].

Notable age and education patterns emerged from our analysis. Younger healthcare professionals (≤30 years) were more likely to belong to the Class 3-Comprehensive Growth Group compared to older professionals (>50 years), with the C2 Skill Recognition Group serving as the reference group (OR = 1.923) in member institutions. Younger healthcare professionals may be more adaptable and open to new models of healthcare [23], such as those introduced by TKUMGs. Additionally, younger professionals are more likely to be in earlier stages of their careers, making them more attuned to opportunities for training and learning [24]. This observation finds support in the Career Construction Theory’s emphasis on adaptability across different career stages. Conversely, healthcare professionals with lower educational levels (college or below) were more likely to experience career development benefits (C3 vs. C1: OR = 3.693 for college or below, OR = 2.260 for undergraduate) compared to those with postgraduate education in member institutions. This may indicate a “ceiling effect”, where highly educated professionals experience limited advancement opportunities within the current TKUMG structure—a phenomenon consistent with the restricted opportunity structures described in the career systems literature [25].

Higher levels of participation (ORs ranging from 1.190 to 1.407) significantly increased the likelihood of healthcare professionals benefiting from the medical group’s development in both leading hospitals and member institutions. This pattern aligns with the principles of Self-Determination Theory, which posits that active involvement in meaningful activities can satisfy individuals’ basic psychological needs for autonomy, competence, and relatedness, thereby enhancing motivation and perceived professional growth [26]. Similarly, positive attitudes were linked to a higher likelihood of benefiting from career development across institutional types. This relationship can be understood through the lens of the Social Exchange Theory: when healthcare professionals perceive the TKUMG as a supportive entity that values their contributions, they are more likely to reciprocate through enhanced engagement and a positive outlook, which in turn facilitates access to developmental resources and opportunities [27]. The interaction between attitudes and benefit attainment appears mutually reinforcing—positive perceptions encourage engagement, and subsequent career benefits further strengthen commitment and trust in the organizational reform.

Departmental affiliations significantly influenced career development trajectories, with the five selected clinical departments and ancillary departments generally showing more favorable outcomes. This variation can be understood through the concept of “institutional centrality” within organizational networks, where certain departments occupy more strategic positions in the TKUMG’s operational framework. This suggests that the career benefits observed for staff in ancillary departments may be due to the standardized internal inspection and verification requirements within the group, along with the integration of information platforms, where departments like electrocardiogram and imaging play an active role. For the five selected clinical departments, the benefits may stem from the need for collaboration across different levels of medical institutions to address common diseases in these specialties, such as diabetes, cardiovascular diseases, and cerebrovascular diseases [28]. From a Social Exchange perspective, these departments may occupy more advantageous positions in the resource exchange network within TKUMGs, receiving greater organizational investment and recognition for their contributions to system-level integration.

## 5. Strengths and Limitations

This study provides insights into the career development of healthcare professionals within TKUMGs, offering a comprehensive analysis based on latent class analysis (LCA) and multinomial logistic regression. One of the key strengths of this study is its inclusion of healthcare professionals from both leading hospitals and member institutions, providing a broad perspective on the varying experiences and benefits within TKUMGs. The use of the LCA allows for the identification of distinct patterns in career development, helping to uncover nuanced factors related to professional growth. Furthermore, this study’s focus on both individual-level factors and institutional factors enhances its relevance for healthcare policymakers and administrators seeking to optimize the TKUMG model. However, several limitations should be considered when interpreting the findings. First, the cross-sectional design precludes causal inferences regarding the relationship between TKUMG participation and career development outcomes. Longitudinal studies would be valuable to examine how career development evolves over time within these groups. Second, in the processing of the latent variables for the LCA model, the workload item was reverse-coded relative to other career development indicators. This may have influenced the analysis and the interpretation of the resultant latent classes. Third, the use of multinomial logistic regression without accounting for the nested structure of the data—where respondents were clustered within six TKUMGs—may have led to underestimated standard errors and inflated Type I error rates. Future studies should employ multilevel multinomial logistic modeling with a random intercept for the TKUMG to appropriately examine cross-level effects. Fourth, this study relies on self-reported data, which is susceptible to common method bias, potentially inflating the observed associations between predictors and outcomes due to consistent response styles or social desirability. Finally, although the sample was drawn from six TKUMGs across two cities, the generalizability of the findings to other regions or healthcare contexts may be limited. Despite these limitations, this study provides valuable preliminary evidence to inform both policy and practice in the development of integrated urban healthcare systems.

## 6. Conclusions

This study identifies three distinct classes of career development benefits among healthcare professionals within TKUMGs, which are shaped significantly by participation levels, personal attitudes, and institutional factors. The findings demonstrate that active engagement and institutional support are essential for achieving comprehensive professional growth. We recommend (1) establishing differentiated incentive policies for member institutions, including special development funds and cross-institutional appointment systems; (2) creating department-specific career pathways, such as vertical specialty alliances for key clinical departments and technical advancement ladders for ancillary departments; and (3) implementing tailored development programs for different demographic groups, including fast-track opportunities for younger professionals and career renewal initiatives for mid-career staff. These targeted strategies will help transform TKUMGs into platforms for more equitable professional growth, ultimately enhancing both workforce satisfaction and system performance.

## Figures and Tables

**Table 1:** Variable assignment.

Variable	Assignment/Value
**Demographics and Professional Background**	
gender	Man = 1, Woman = 2
age	≤30 = 1, 31–40 = 2, 41–50 = 3, >50 = 4
education	Junior College and Below = 1, Bachelor’s Degree = 2, Graduate Degree and Above = 3
professional title	None = 1, Junior = 2, Intermediate = 3, Senior = 4
affiliated TKUMG ^1^	High-Score Group = 1, Medium-Score Group = 2, Low-Score Group = 3
affiliated institution	Leading Hospital = 1, Member Institution = 2
affiliated department	The Five Selected Clinical Departments = 1, General = 2, Ancillary = 3, Other = 4
**Participation and Attitudes Toward the TKUMG ^1^**	
individual attitude	10-Point Scale
individual participation level	
**Perceived Changes in Personal Development**	
compensation level	“Decrease” = 1, “No Change” = 2, “Increase” = 3
workload	
professional title advancement opportunities	
training availability	
patient recognition and trust	
utilization of one’s skills and expertise	

^1^ TKUMGs = tightly knit urban medical groups.

**Table 2:** The comprehensive evaluation score of the construction of the six TKUMGs ^1^ in four dimensions (*n* = 6).

TKUMG ^1^	Score in the Dimension of Authority and Responsibility Coordination	Score in the Dimension of Resource Coordination	Score in the Dimension of Business Coordination	Score in the Dimension of Mechanism Coordination	Final Comprehensive Score
H1 ^2^	3.33	1.88	4.44	10.00	19.65
H2 ^2^	1.67	1.25	4.44	0.00	7.36
H3 ^2^	1.67	1.09	1.11	0.00	3.87
Q1 ^3^	3.33	2.23	10.00	0.00	15.56
Q2 ^3^	6.67	3.79	7.78	10.00	28.24
Q3 ^3^	8.33	3.69	10.00	0.00	22.02

^1^ TKUMGs = tightly knit urban medical groups. ^2^ H1, H2, and H3 are the simple anonymous terms for the three pilot TKUMGs located in Qiqihar. ^3^ Q1, Q2, and Q3 are the simple anonymous terms for the three pilot TKUMGs located in Hefei. Note: each dimension of the evaluation score was rated on a 10-point scale, and the final comprehensive score was the sum of four dimensions, with a full score of 40 points.

**Table 3:** Demographic characteristics of the participants (*n* = 2200).

Variable	Leading Hospital (*n* = 942)	Member Institution (*n* = 1258)	*p*-Value
**Male, *n* (%)**	276 (29.3)	366 (29.1)	0.916
**Age, *n* (%)**			<0.001
≤30	158 (16.8)	163 (13.0)	
31–40	487 (51.7)	505 (50.9)	
41–50	232 (24.6)	442 (35.1)	
>50	65 (6.9)	148 (11.8)	
**Education, *n* (%)**			<0.001
Junior College and Below	62 (6.6)	336 (26.7)	
Bachelor’s Degree	594 (63.1)	838 (66.6)	
Graduate Degree and Above	286 (30.4)	84 (6.7)	
**Professional Title, *n* (%)**			<0.001
None	36 (3.8)	93 (7.4)	
Junior	245 (26.0)	380 (30.2)	
Intermediate	408 (43.3)	537 (42.7)	
Senior	253 (26.9)	248 (19.7)	
**TKUMG ^1^, *n* (%)**			<0.001
H1 ^2^	165 (17.5)	263 (20.9)	
H2 ^2^	349 (37.0)	401 (31.9)	
H3 ^2^	153 (16.2)	352 (28.0)	
Q1 ^3^	183 (19.4)	142 (11.3)	
Q2 ^3^	89 (9.4)	77 (6.1)	
Q3 ^3^	3 (0.3)	23 (1.8)	
**Institution Department, *n* (%)**			<0.001
The Five Selected Clinical Departments	598 (63.5)	183 (14.5)	
General	60 (6.4)	473 (37.6)	
Ancillary	247 (26.2)	96 (7.6)	
Other	37 (3.9)	506 (40.2)	
**Individual Attitude, M (Q1, Q3) ** ^4^	10 (10, 10)	10 (8.75, 10)	<0.001
**Individual Participation level, M (Q1, Q3) ** ^4^	5 (2.5, 8.75)	3.75 (1.25, 7.5)	<0.001

^1^ TKUMGs = tightly knit urban medical groups. ^2^ H1, H2, and H3 are the simple anonymous terms for the three pilot TKUMGs located in Qiqihar. ^3^ Q1, Q2, and Q3 are the simple anonymous terms for the three pilot TKUMGs located in Hefei. ^4^ M = Median, Q1 = the first quartile, and Q3 = the third quartile.

**Table 4:** Performance of latent class analysis models (*n* = 2200).

Number of Classes	AIC ^2^	BIC ^2^	aBIC ^2^	Entropy	BLRT ^2^	VLMR ^2^	Minimum Class Population Ratio
1	20,106.81	20,175.17	20,137.04				
2	16,092.21	16,234.62	16,155.19	0.853	<0.001	<0.001	48.3%
**3 ^1^**	**15,252.99**	**15,469.45**	**15,348.71**	**0.881**	**<0.001**	**<0.001**	**32.0%**
4	14,772.48	15,062.99	14,900.95	0.898	<0.001	<0.001	3.5%
5	14,729.71	15,094.27	14,890.93	0.886	0.0261	0.0251	1.3%

^1^ Model 3 was chosen due to the balanced number of people in each class, low BIC value (15,469.45), high entropy value (0.881 > 0.800), significant BLRT and VLMR (*p* < 0.001). ^2^ Note: the *p*-value is reported for the BLRT and VLMR. Abbreviations: AIC = Akaike Information Criterion; BIC = Bayesian Information Criterion; aBIC = Sample-Size-Adjusted BIC; BLRT = Bootstrap Likelihood Ratio Test; and VLMR = Vuong–Lo–Mendell–Rubin Test.

## Data Availability

The dataset used and analyzed in this study is available from the corresponding author. The data are not publicly available due to privacy concerns.

## Abbreviations

The following abbreviations are used in this manuscript:

TKUMGs	Tightly knit urban medical groups
LCA	Latent class analysis

## Appendix A

### Appendix A.1. Details of the Comprehensive Evaluation Score for the TKUMG’s Construction

In the comprehensive evaluation score for the TKUMG’s construction, 1 point will be awarded if the result of each item is ‘‘yes”, ‘‘all interconnected”, or ‘‘all shared”; 0 points will be awarded if the result of each item is ‘‘no”, ‘‘not interconnected”, or ‘‘not shared”; and 0.5 points will be awarded if the result of each item is ‘‘partial interconnected” or ‘‘partial shared”. When calculating the final comprehensive score, the weight of each dimension is the same. Under the second dimension, resource coordination, there are four aspects with the same weight. Ultimately, after standardizing the scoring system, each dimension was constructed with a 10-point scale, and the final score was added up to 40 points.

**Table A1:** This is a table that shows the specific items of each scoring dimension of the TKUMG ^1^’s comprehensive evaluation score and the results for each TKUMG.

	TKUMG	H1 ^2^	H2 ^2^	H3 ^2^	Q1 ^3^	Q2 ^3^	Q3 ^3^
Item							
1. Dimension of Authority and Responsibility Coordination							
1.1. Whether to establish a management committee with the participation of relevant local government departments and the TKUMG.		○	○	○	○	○	○
1.2. Whether the autonomy of the internal institutions of the TKUMG has been implemented.		○	×	×	×	○	○
1.3. Whether the autonomy in the job setting for the TKUNMG has been implemented.		×	×	×	×	○	○
1.4. Whether the autonomy of the professional title appointment for the TKUMG has been implemented.		×	×	×	×	×	×
1.5. Whether the autonomy in the selection and appointment of the TKUMG has been implemented.		×	×	×	×	×	○
1.6. Whether the autonomy in the performance distribution within the TKUMG has been implemented.		×	×	×	○	○	○
**2. Dimension of Resource Coordination**							
**2.1. Integrated management of staff**							
2.1.1. Whether unified recruitment is implemented for staff within the TKUMG.		×	×	×	×	×	×
2.1.2. Whether the staff within the TKUMG are subject to unified assessment.		×	×	×	×	×	×
2.1.3. Whether the staff within the TKUMG are used in a coordinated manner.		×	×	×	×	×	○
**2.2 Integrated financial management**							
2.2.1. Whether to set up a financial management center.		○	×	×	×	○	×
2.2.2. Whether to coordinate the budget management of the TKUMG.		×	×	×	×	○	○
2.2.3. Whether to coordinate the asset management of the TKUMG.		×	×	×	×	×	×
2.2.4. Whether to coordinate the accounting supervision of the TKUMG.		×	×	×	○	○	×
**2.3. Integrated management of materials**							
2.3.1. Whether to unify the drug management platform.		×	×	×	×	×	×
2.3.2. Whether to unify the consumables management platform.		×	×	×	×	×	×
2.3.3. Whether to unify the large-scale equipment management platform.		×	×	×	×	×	×
2.3.4. Whether the drug formulary is integrated across institutions.		○	○	○	×	×	×
2.3.5. Whether the integrated distribution and payment for drug procurement have been achieved.		×	×	×	×	×	×
2.3.6. Whether the integrated distribution and payment for consumables procurement have been achieved.		×	×	×	×	×	×
2.3.7. Whether the integrated distribution and payment for large-scale equipment procurement have been achieved.		×	×	×	×	×	×
**2.4. Information interconnection and interoperability as well as the joint construction and sharing of high-quality resources**							
2.4.1. Interconnection of management information within the consortium.		⛒	⛒	⛒	⛒	⛒	○
2.4.2. Interconnection of medical records within the consortium.		⛒	⛒	⛒	⛒	⛒	○
2.4.3. Interconnection of patient information within the consortium.		⛒	⛒	⛒	⛒	⛒	○
2.4.4. Coordination in the development of shared medical laboratory centers.		⛒	⛒	⛒	⛒	⛒	○
2.4.5. Coordination in the development of shared medical imaging centers.		⛒	⛒	⛒	⛒	○	○
2.4.6. Coordination in the development of shared electrocardiogram (ECG) diagnostic centers.		⛒	⛒	×	⛒	⛒	×
2.4.7. Coordination in the development of shared pathology resource centers.		⛒	⛒	⛒	⛒	○	×
2.4.8. Coordination in the development of centralized disinfection and supply resource centers.		⛒	⛒	⛒	⛒	⛒	○
**3. Dimension of Business Coordination**							
3.1. Whether to unify the integrated management of medical quality and safety.		○	⛒	⛒	○	○	○
3.2. Whether to unify the integrated management of hospital infection control.		⛒	⛒	⛒	○	○	○
3.3. Whether to unify the integrated management of medical record quality.		⛒	⛒	⛒	○	○	○
3.4. Whether to unify the integrated management of prescription circulation.		⛒	⛒	⛒	○	⛒	○
3.5. Whether to unify the integrated management of disease prevention and control.		⛒	⛒	⛒	○	⛒	○
3.6. The participation of physicians from secondary and tertiary hospitals in family physician contract service teams.		○	○	○	○	○	○
3.7. Implementation of collaborative mechanisms with public health professionals.		○	○	⛒	○	○	○
3.8. Establishment of information coordination with specialized public health institutions.		⛒	○	⛒	○	○	○
3.9. Collaboration with specialized public health institutions.		○	○	⛒	○	○	○
**4. Dimension of Mechanism Coordination**							
4.1. Existence of financial support for the compact urban medical consortium.		○	○	×	○	○	○

^1^ TKUMGs = tightly knit urban medical groups. ^2^ H1, H2, and H3 are the simple anonymous terms for the three pilot TKUMGs located in Qiqihar. ^3^ Q1, Q2, and Q3 are the simple anonymous terms for the three pilot TKUMGs located in Hefei. Note: “○” = Yes, all interconnected, all shared; “×” = No, not interconnected, not shared; “⛒” = Partial interconnected, partial shared.

### Appendix A.2. Results of Univariate Analysis

The three latent classes identified from the latent class analysis were used as the dependent variables, and gender, age, education, professional title, affiliated TKUMG, institution, department, individual attitude, and individual participation were the independent variables for univariate analysis.

**Table A2:** Testing for differences between the studies’ participants classified different groups regarding gender, age, education, professional title, affiliated TKUMG ^1^, institution, department, individual attitude, and individual participation.

Variable/Category	Total (*n* = 2200)	Class 1 ^5^ (*n* = 712)	Class 2 ^5^ (*n* = 783)	Class 3 ^5^ (*n* = 705)	Teat Statistic	*p*-Value
**Gender, *n* (%)**					χ^2^ = 1.27	0.53
Male	642 (29.2)	202 (28.4)	240 (30.7)	200 (28.4)		
Female	1558 (70.8)	510 (71.6)	543 (69.3)	505 (71.6)		
**Age, *n* (%)**					χ^2^ = 39.779	<0.001
≤30	321 (14.6)	99 (13.9) a	83 (10.6) a	139 (19.7) b		
31–40	992 (45.1)	324 (45.5) a	337 (43.0) a	331 (47.0) a		
41–50	674 (30.6)	226 (31.7) a	270 (34.5) a	178 (25.2) b		
>50	213 (9.7)	63 (8.8) a, b	93 (11.9) b	57 (8.1) a		
**Education, *n* (%)**					χ^2^ = 21.274	<0.001
Junior College and Below	398 (18.1)	153 (21.5) a	148 (18.9) a	97 (13.8) b		
Bachelor’s Degree	1432 (65.1)	462 (64.9) a	504 (64.4) a	466 (66.1) a		
Graduate Degree and Above	370 (16.8)	97 (13.6) a	131 (16.7) a, b	142 (20.1) b		
**Professional Title, *n* (%)**					χ^2^ = 17.682	0.007
Junior College and Below	129 (5.9)	49 (6.9) a	40 (5.1) a	40 (5.7) a		
Bachelor’s Degree	625 (28.4)	209 (29.4) a, b	196 (25.0) b	220 (31.2) a		
Graduate Degree and Above	945 (43.0)	319 (44.8) a	349 (44.6) a	277 (39.3) a		
Junior College and Below	501 (22.8)	135 (19.0) a	198 (25.3) b	168 (23.8) a, b		
**TKUMG ^1^, *n* (%) (Group according to the TKUMG’s comprehensive evaluation scores ^6^)**					χ^2^ = 95.745	<0.001
High-Score Group (Q2 ^3^, Q3 ^3^)	1225 (57.0)	444 (62.4) a	441 (56.3) a, b	370 (52.5) b		
Medium-Score Group (H1 ^2^, Q1 ^3^)	753 (34.2)	235 (33.0) a, b	303 (38.7) b	215 (30.5) a		
Low-Score Group (H2 ^2^, H3 ^2^)	192 (8.7)	33 (4.6) a	39 (5.0) a	120 (17.0) b		
**Institution, *n* (%)**					χ^2^ = 35.812	<0.001
Leading Hospital	942 (42.8)	259 (36.4) a	319 (40.7) a	364 (51.6) b		
Member Institution	1258 (57.2)	453 (63.6) a	464 (59.3) a	341 (48.4) b		
**Institution Department, *n* (%)**					χ^2^ = 248.072	<0.001
The Five Selected Clinical Departments	781 (35.5)	157 (22.1) a	223 (28.5) b	401 (56.9) c		
General	533 (24.2)	191 (26.8) a	230 (29.4) a	112 (15.9) b		
Ancillary	543 (24.7)	109 (15.3) a	129 (16.5) a	105 (14.9) a		
Other	343 (15.6)	255 (35.8) a	201 (25.7) b	87 (12.3) c		
**Individual Attitude,** **M (Q1, Q3) ** ^4^	10.00 (10.00, 10.00)	10.00 (7.50, 10.00)	10.00 (10.00, 10.00)	10.00 (10.00, 10.00)	Hc = 320.135	<0.001
**Individual Participation Level,** **M (Q1, Q3) ** ^4^	5.00 (2.50, 8.75)	2.50 (0.00, 5.00)	5.00 (2.50, 7.50)	8.75 (5.00, 10.00)	Hc = 423.853	<0.001

^1^ TKUMGs = tightly knit urban medical groups. ^2^ H1, H2, and H3 are the simple anonymous terms for the three pilot TKUMGs located in Qiqihar. ^3^ Q1, Q2, and Q3 are the simple anonymous terms for the three pilot TKUMGs located in Hefei. ^4^ M = median, Q1 = the first quartile, and Q3 = the third quartile. ^5^ C1 = C1-Limited Growth Group, C2 = C2-Skill Recognition Group, and C3 = C3-Comprehensive Growth Group. ^6^ TKUMG’s score: the score of the overall assessment of TKUMG development above, and it is divided into high-score group, medium-score group, and low-score group according to the score. Note: Different letters (a, b, c) following values indicate statistically significant differences among groups (*p* < 0.05). Groups that do not share a common letter are significantly different from each other.
